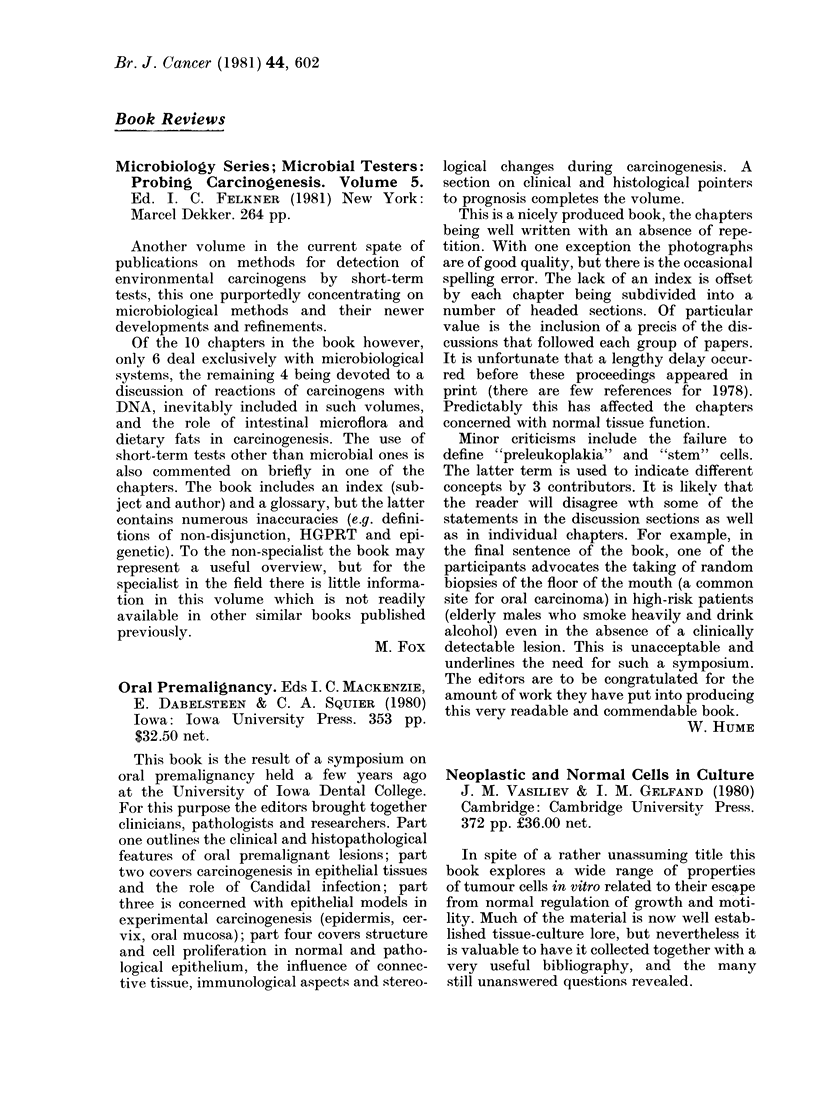# Microbiology Series; Microbial Testers: Probing Carcinogenesis. Volume 5

**Published:** 1981-10

**Authors:** M. Fox


*Br. J. Cancer* (1981) **44**, 602

### *Book Reviews*

**Microbiology Series; Microbial Testers: Probing Carcinogenesis. Volume 5.** Ed. I. C. Felkner (1981) New York: Marcel Dekker. 264 pp.

Another volume in the current spate of publications on methods for detection of environmental carcinogens by short-term tests, this one purportedly concentrating on microbiological methods and their newer developments and refinements.

Of the 10 chapters in the book however, only 6 deal exclusively with microbiological systems, the remaining 4 being devoted to a discussion of reactions of carcinogens with DNA, inevitably included in such volumes, and the role of intestinal microflora and dietary fats in carcinogenesis. The use of short-term tests other than microbial ones is also commented on briefly in one of the chapters. The book includes an index (subject and author) and a glossary, but the latter contains numerous inaccuracies (*e.g.* definitions of non-disjunction, HGPRT and epigenetic). To the non-specialist the book may represent a useful overview, but for the specialist in the field there is little information in this volume which is not readily available in other similar books published previously.

M. Fox